# The effect of cognitive functional therapy for chronic nonspecific low back pain: a systematic review and meta-analysis

**DOI:** 10.1186/s13030-022-00241-6

**Published:** 2022-05-21

**Authors:** Takahiro Miki, Yu Kondo, Hiroshi Kurakata, Eva Buzasi, Tsuneo Takebayashi, Hiroshi Takasaki

**Affiliations:** 1 Sapporo Maruyama Orthopedic Hospital, N7 W 27 Chuo Hokkaido, Sapporo, 006-0007 Japan; 2 Faculty of Health Sciences, Hokkaido University, Sapporo, Japan; 3 Graduate school, Saitama Prefectural University, Koshigaya, Saitama Japan; 4 Yumenomachi Home Nursing Care and Rehabilitation Service, Chiba, Japan; 5 Division of Surgery and Interventional Science, University College London, London, UK; 6 Department of Physical Therapy, Saitama Prefectural University, Koshigaya, Saitama Japan

**Keywords:** Cognitive functional therapy, Chronic nonspecific low back pain, Fear of physical activity

## Abstract

**Background:**

To apply the Bio-Psych-Social (BPS) model into clinical practice, it is important not to focus on psychosocial domains only since biomedical factors can also contribute to chronic pain conditions. The cognitive functional therapy (CFT) is the management system based on the BPS model for chronic nonspecific low back pain (CNSLBP).

**Objectives:**

This study aimed to compare CFT with the other interventions for CNSLBP regarding pain, disability/functional status, QoL and psychological factors.

**Design:**

This study was a systematic review and meta-analysis of a randomised controlled trial.

**Method:**

Literature Search was conducted in electronic search engines. Enrolled participants included 1) CNSLBP and 2) primary, secondary, or tertiary care patients. CFT was the interventions included. Comparisons were any types of treatment.

**Results:**

Three studies met the eligibility criteria. The total number of participants was 336. For pain intensity, MD [95% CIs] was -1.38 [-2.78 − 0.02] and -1.01 [-1.92 − -0.10] at intermediate and long term for two studies, respectively. About disability/functional status, SMD [95% CIs] was -0.76 [-1.46 − -0.07] at the intermediate for three studies and MD [95% CIs] was -8.48 [-11.47 − -5.49] at long term for two studies. About fear of physical activity, MD [95% CIs] was -3.01 [-5.14 − -0.88] and -3.56 [-6.43 − -0.68] at intermediate and long term for two studies, respectively. No studies reported scores associated with QOL. All the quality of the evidence was very low.

**Conclusions:**

Three studies were included and the quality of all the evidence was very low. Although the study found statistically significant differences in some measures, the effectiveness of the CFT will need to be re-evaluated in the future.

**Trial registration:**

PROSPERO registration number CRD42020158182.

**Supplementary Information:**

The online version contains supplementary material available at 10.1186/s13030-022-00241-6.

## Background

The Bio-Psych-Social (BPS) model of care is recommended in clinical practice guidelines for chronic nonspecific low back pain (CNSLBP) [1]. However, implementing the BPS approach for CNSLBP has not been conducted well by physical therapists [2]. One reason is the lack confidence in the interventions based on the BPS model [3]. To apply the BPS model in clinical practice, it is important to not focus only on psychosocial domains because biomedical factors can also contribute to chronic pain conditions [4]. Cognitive Behavioral Therapy (CBT) is effective for CNSLBP but only to some extent [5]. In this study, there was a small significant effect in pain for both short-term (SMD [95% CIs] = 0.26 [0.41, 0.11]) and long-term (SMD [95% CIs] = 0.21 [0.33, 0.09]) effects. However, for disability, only the long-term effect was significant (SMD [95% CIs] = 0.19 [0.32, 0.07]), and there was no significant effect for QOL in either the short or long term [5]. In addition, CBT is limited in that it is a psychological approach and not a BPS approach [6]. This characteristic of the CBT may reflect the facts that physical therapists reported the lack of CBT implementation into clinical practice due to limited knowledge about CBT techniques [3]. Furthermore, several the physical therapists mentioned that the CBT components were not parts of physical therapy [3]. On the other hand, the approach based on the BPS model is a multifaceted intervention that considers all biomedical, psychological, and social factors. These three factors interact with each other to influence pain and disability. Therefore, a multidisciplinary intervention like the BPS approach may be able to improve disability and QoL, which CBT does not improve.

Recently, Peter O’Sullivan and colleagues introduced Cognitive Functional Therapy (CFT) [7]. CFT is an example of physical therapists using the BPS approach for CNSLBP and is increasingly of interest. While CBT aims to improve psychological factors by identifying and changing maladaptive thought patterns, behaviors, and environments [6], CFT aims to enable the therapist to lead the patient to effectively perform self-management using the three primary components; "Making sense of pain", "Exposure with control", and "Lifestyle change" (See Additional file 1). This is similar to the concept of CBT in some aspects. For instance, “Making sense of pain" in CFT corresponded to psychoeducation and relaxation in CBT, "Exposure with control" to interoceptive exposure in CBT, and "lifestyle change" corresponded to behavioral activation in CBT. On the other hand, the differences between CBT and CFT are that CBT improves pain management and coping mechanisms from a mental approach only while CFT directly addresses maladaptive behaviours and uses cognitively integrated progressive load to encourage patients to improve their functioning actively [6]. CBT is fundamentally a treatment strategy for primary mental disorders and does not generally improve movement or function. In addition, CBT targets many mental illnesses, depression, anxiety, stress, and chronic pain, while CFT is preferred in musculoskeletal disorders, especially CNSLBP, where physical therapists play a leading role [6]. Details of the CFT have been explained in detail in a previous study [7]. Briefly, the CFT helps CNSLBP patients understand their pain features and develops an individually tailored management strategy. The CFT uses a multidimensional clinical reasoning framework [8] and identifies key modifiable targets for management in each patient through careful listening to their individual story, and experiential learning on responses to pain by challenging expectations of pain in guided behavioural experiments, which has components of a exposure-based approach. Using an exposure-based approach is effective with individuals who have a strong level of fear, which is one factor that can lead to a poor prognosis in patients with low back pain (LBP) [9].

CFT can be a promising system based on the BPS model of care in managing CNSLBP, resulting in may improve disability and QOL as well as pain intensity [7]. However, it is not known whether CFT is more effective than usual care for CNSLBP patients and how much overall quality of evidence exists in the effectiveness of the CFT due to the lack of data synthesis. This systematic summary of the effectiveness of CFT, an approach based on the BPS model, will be one of the ways to build evidence for the effectiveness of future approaches based on the BPS model. In order to summarize the evidence, this systematic review with meta-analysis of randomized controlled trials (RCTs) aimed to evaluate the effectiveness of the CFT by comparing the CFT and the other treatments of pain, disability, quality of life, and psychological status among individuals with CNSLBP. The primary outcome measures were disability, pain intensity, and QOL.

## Materials and methods

### Protocol and registration

This systematic review was pre-registered in the Prospero Database (CRD42020158182) and followed the Preferred Reporting Items for Systematic Reviews and Meta-Analyses (PRISMA) guidelines [10].

### Information sources and search

One author (HK) systematically searched the following databases from inception to Dec 2020: Web of science, CENTRAL, MEDLINE, EMBASE, PsycINFO, EMCARE, CINAHL, AMED and PEDro databases. Although CENTRAL and AMED were not listed in the PROSPERO, a more accurate search was performed. The search strategies were presented in Additional file 2 (See Additional file 2). Moreover, cross-referencing was done to the primary developer of the CFT, Peter O’Sullivan, and relevant literatures cited in the studies were searched manually.

### Eligibility criteria

The PICOS framework was used in developing the eligibility criteria. Enrolled participants included 1) patients with CNSLBP; 2) patients from primary, secondary, or tertiary care; and 3) patients without radiating pain to lower extremities. Studies with the following participants were not included in this systematic review: 1) participants with LBP caused by serious pathologies, including infections, neoplasms, metastases, fractures, osteoporosis, rheumatoid arthritis, and radiculopathies; 2) participants with LBP during or immediately following pregnancy; and 3) participants with postoperative back pain. Eligible interventions include the CFT as both managements were conceptually comparable, which was confirmed by the primary developer, Peter O’Sullivan. Eligible comparisons included any types of interventions other than the CFT. Eligible outcomes included pain intensity, back-specific disability/function status, quality of life, and psychological status. Eligible study design was only the published RCTs although it included observational studies in PROSPERO.

### Study selection and data collection process

Screening and full-text inspection were performed by two authors (TM and YK) independently, where publication source, authors, and publication year were not blinded. The screening was based on the information in the title and abstract. Any disagreements on eligibility were resolved through a consensus between the two authors.

### Risk of bias in individual studies

The risk of bias was assessed using a 10-point PEDro score, which was changed from the Cochrance (RoB) registered in the initial draft of the PROSPERO. This is because the PEDro score was specifically developed to assess the risk of bias of physical therapy trials [11, 12], and established scores are presented. Inclusion criteria for the meta-analysis was high-quality study, which was defined as the study with a PEDro score of 6 or higher [13, 14].

### The quality of the evidence

The overall quality of evidence for each meta-analysis was identified using the Grading of Recommendations Assessment, Development and Evaluation (GRADE) system [15] as recommended by the Updated Method Guideline for Systematic Reviews in the Cochrane Back and Neck Group [16]. The GRADE system consists of five items: 1) risk of bias, 2) inconsistency, 3) indirectness, 4) imprecision, and 5) publication bias. Each criterion was rated as high, moderate, low, or very low, and the lowest quality was chosen as the overall quality of evidence. For evidence from RCTs, we started with a rating of `high`. The quality of evidence on specific outcomes was reduced by one or two levels depending on the performance of specific comparative studies on these five factors. Regarding the risk of bias, the quality of the evidence has been reduced by one point if more than 25% and two points if more than 50% of the participants were from studies conducted in low-quality methods (e.g., PEDro score < 6). For inconsistency, the quality of evidence has been reduced by one point when the heterogeneity or variability in results was large, as indicated by an I^2^ > 50% and reduced by two points when an I^2^ > 75%. For indirectness, whether the issues addressed in this systematic review differed from the available evidence on populations, interventions, comparisons and outcomes has been assessed. One points was deducted if there was indirectness in only one area and two points if there was indirectness in two or more areas. As regards imprecision, one point was deducted if the total number of participants was less than 400. In addition, if there was no significant difference in outcomes, the grade was reduced. Regarding publication bias, a funnel plot was created to compare at least ten studies, and from the funnel plot, the quality of the evidence was reduced by one point if publication bias was suggested.

The two authors (TM and YK) independently assessed GRADE scores in each meta-analysis, and any disagreements were resolved by another author (HK).

### Data items and summary measures

Data were extracted based on the PICOS framework by the two authors (TM and YK) with consensus. Extracted data of the participants included participant source and setting, age, gender, and duration of symptoms. Extracted data of the intervention included description of interventions, duration and number of sessions, therapist training level on the CFT, and delivery type such as individual or group. Extracted data of comparisons included type of intervention and duration and/or number of sessions. Extracted data of outcomes included means and SDs of pain, disability/function status, quality of life, and psychological factors during short, intermediate- and/or long-term follow-ups. In this study, the short term was defined as post-treatment. The intermediate term was defined as ≥ 3 months and < 12 months, and the long term was defined as ≥ 12 months [17]. In the presence of more eligible follow-up points, the follow-up point closest to 6 months was chosen for the intermediate term and the follow-up point closest to 12 months was chosen for the long term. The meta-analysis was attempted using change values from the baseline to each follow-up point first [18]. If there was no change values reported in the study, a corresponding author was contacted by email and was asked to provide the data. If the value of the change was not available, the values at each follow-up point was used for meta-analysis [18]. Data on serious adverse reports were also extracted, including serious accidents and deaths during the intervention. Extracted data of the study design included country of data collection and source of research grant.

### Synthesis of results and statistical analysis

When multiple datasets of similar outcomes were present, meta-analysis was performed using Review Manager 5 (The Nordic Cochrane Centre, København Ø, Denmark). The mean difference (MD) or the standardised mean difference (SMD) with 95% confidence intervals (95% CI) was calculated using the random-effect model. The MD was calculated when similar outcomes were assessed with similar patient-reported outcomes measures, and the SMD was used when similar outcomes were assessed with different patient-reported outcomes measures. The I^2^ statistic was assessed for heterogeneity among trials, whose interpretations were as follows: 0 − 40% = may be insignificant, 30 − 60% = moderate heterogeneity, 50 − 90% = substantial heterogeneity, and 75 − 100% = considerablel heterogeneity [18]. The sensitivity analysis was undertaken when considerable heterogeneity existed and thus the sensitivity analysis was possible.

## Results

### Study selection

**Fig. 1:**
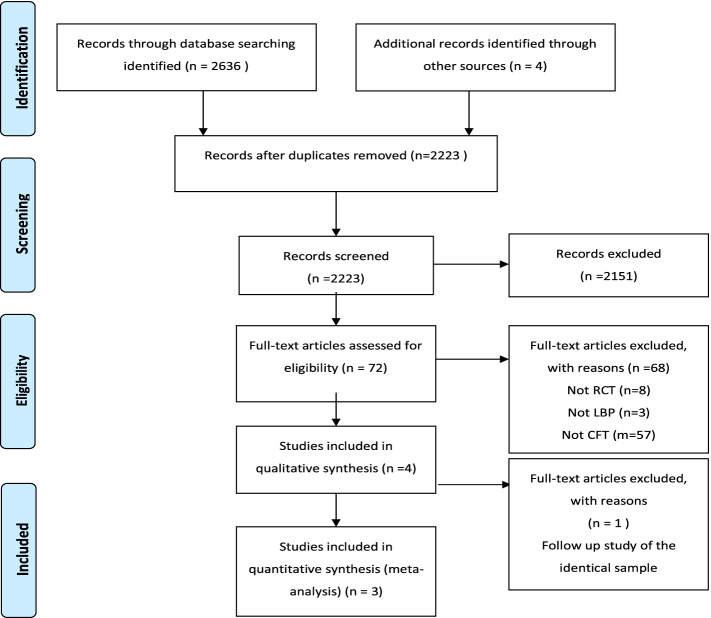
Flow diagram of search strategy

Figure 1 presents the flow of the study selection. Disagreement rate between the two authors in the screening process and in the full-text inspection process was 15.5% and 4.2%, respectively. Four studies [19–22] were eligible. Two studies [19, 22] were from the same study project. However, one study [22] was excluded since the data reported were from the same subjects at a 3-year follow-up and no additional data were found that were eligible for extraction in the current systematic review. Consequently, three studies [19–21] were included the systematic review and assessed for the risk of bias.

### Study characteristics

The three eligible studies [19–21] are summarised in Table 1. All studies were RCTs performed by the same research group. All studies reported pain, where in one study reported change in pain intensity during 15 min of rowing and other studies reported average pain intensity over a week using the 11-point numerical rating scale. Therefore, the study was not included in the meta-analysis [20]. The other two studies of pain intensity outcomes were considered to have similar patient-reported outcomes measures allowing meta-analysis with the MD [19, 21]. All studies reported back-specific disability/functions status: one study used Roland-Morris Disability Questionnaire (RMDQ), and the other two studies used the Oswestry Disability Index (ODI). RMDQ and ODI were considered to have similar outcomes allowing meta-analysis with the SMD. Regarding psychological status, two studies reported fear of physical activity using the Fear-Avoidance Beliefs Questionnaire (FABQ), and meta-analysis was performed with the MD. No studies reported scores associated with quality of life. None of the studies reported serious adverse reports.

**Table 1:** Summary of the three studies included in the meta-analysis

Study, data collection country, and the source of funding	Participants	Interventions	Comparisons	Outcome measures
Fersum [19] Norway, The Norwegian Fund	Total *n* = 94 (from private physiotherapy outpatient practices, general practitioners, and the outpatient spine clinic at the Haukeland University Hospital in Bergen). Participants were aged between 18 and 65 years old diagnosed with nonspecific chronic low back pain for > 3 months CFT group: Age 41 ± 10.3 years. Gender 24 males, 27 females. Mean duration of symptoms 3–12 months = 6, 1–5 years = 14, > 5 years = 31 Control group: Age 42.9 ± 12.5 years. Gender 22 males, 21 females. Mean duration of symptoms 3–12 months = 6, 1–5 years = 13, > 5 years = 23	Individual Intervention. CFT for 12 weeks by three experienced physiotherapists who had undergone, on average, 106 h of CB-CFT training. The mean number of treatments was 7.7 ± 2.6. The initial session was 1 h, and follow-ups were 30–45 min	Individual Intervention. Joint mobilisation or manipulation techniques for 12 weeks by therapists who were specialists in orthopaedic manual therapy with an average of 25.7 years of experience with no prior training in the use of the MDCS or CB-CFT. Most patients (82.5%) were also given exercises, which included general or motor control exercise as a home programme. The mean number of treatments was 8.0 ± 2.9. The initial session was 1 h, and follow-ups were 30 min	Pain: Week average, NRS Disability/functional status: ODI Fear physical activity: FABQ Other objective measures that were not included in this review Anxiety and Depression: HSCL-25 Lumbar ROM: Inclinometer method Patient satisfaction: PSQ Sick-leave days: OMPQ Follow-up: 3, 12 months
Ng [20] Australia, Sports Physiotherapy Australia grant	Total *n* = 36 (from school and community rowing clubs in Perth, Western Australia). Participants were adolescent male rowers aged between 14 and 19 years old with between 1 and 4 years of school-level rowing experience, suffering from LBP related to rowing CFT group: Age 16.3 ± 1.5 years. Gender 19 males Control group Age 15.2 ± 1.5 years. Gender 17 males	Individual Intervention. CFT for 8 weeks by a physical therapist with 5 years’ experience in the Australian Rowing and training in CFT. The initial session was approximately 1 h in duration and follow-up appointments were 30 min. Rowers were seen a week after the initial session and then fortnightly after that	The control group did not receive any elements of the CFT intervention from their coaches or the treating physiotherapist	Disability/functional status: RMDQ Other objective measures that were not included in this review Pain: Mean maximum pain during a 15-min ergometer trial, NRS Back and lower limb muscle endurance: Biering-Sorensen test and isometric squat test Lumbar kinematics: upper and lower lumbar angle during 15-min ergometer Follow-up: 8, 12 weeks
O’Keeffe [21] Australia, No funding	Total: n = 206 (from Ballina Primary Care Centre, Claremorris Primary Care Centre and Mayo General Hospital) The participants were between 18 and 75 years of age, CNSLBP for at least 6 months duration CFT group: Age 47.0 ± 13.2 years. Gender 24 males, 82 females. Median duration of symptoms 56 (24–120) months Control group: Age 50.6 ± 14.9 years. Gender 30 males, 70 females. Median duration of symptoms: 60 (24–156) months	Individual Intervention. CFT for 12 weeks by three experienced physiotherapists who had undergone of CB-CFT training. Treatment was given weekly and reduced in frequency over time. The initial session was 1 h, and follow-ups were 30–60 min	Group-based exercise and education intervention consisting of up to six classes over 6–8 weeks, each lasting ~ 1 h and 15 min, with up to 10 participants in each class	Pain: Week average, NRS Disability/functional status: ODI Fear physical activity: FABQ Other objective measures that were not included in this review Beliefs: BBQ Self-efficacy: PSEQ Coping: CSQ Sleep, depression and anxiety: SHC Stress: DASS Patient satisfaction: PSQ Follow-up: Post intervention, 6, 12 months

*CFT* cognitive functional therapy*, NRS* Numerical Rating Scale, *ODI* Oswestry Disability Index, *FABQ* Fear-Avoidance Beliefs Questionnaire, *MDCS* Multidimensional Classification System, *ROM* Range of Motion, *RDQ* Roland-Morris Disability Questionnaire, *HSCL-25* Hopkins Symptoms Checklist, *PSQ* Patient Satisfaction Questionnaire, *OMPQ* Orebro Musculoskeletal Pain Questionnaire, *CNSLBP* chronic non-specific low back pain, *BBQ* Back Beliefs Questionnaire, *PSEQ* Pain Self-Efficacy Questionnaire, *CSQ* Coping Strategies Questionnaire, *SHC* Subjective Health Complaints Inventory, *DASS* Depression, Anxiety and Stress Scale

### Risk of bias within studies

As all studies included in this systematic review [19–21] were found in the PEDro online database, the scores from the PEDro online database were used. The quality scores of the three eligible studies [19–21] are summarized in Table 2. For Ng et al.’s study [20], the sample size was not large, and the participants were somewhat biased toward rowing athletes. Ng and colleagues did not perform an intention-to-treat analysis and evaluated pain immediately after exercise using a somewhat unique method. O’Keeffe et al.’s study [21] was not blinded, and many participants dropped out during the follow-up. Fersum et al.’s study [19] did not conduct an intention-to-treat analysis and did not have sufficient blinding. In particular, there may be a substantial violation of the intention-to-treat analysis because of the exclusion of 27/121 patients before the 3-month follow-up [23].

**Table 2:** PEDro scores of included studies

Study	Item1	Item2	Item3	Item4	Item5	Item6	Item7	Item8	Item9	Item10	Item11	Total (0 to 10)
Fersum	Y	Y	Y	Y	N	N	N	N	N	Y	Y	5
Ng	Y	Y	Y	Y	N	N	Y	Y	N	Y	Y	7
O’Keeffe	Y	Y	Y	Y	N	N	N	N	Y	Y	Y	6

*Item1* Eligibility criteria (not scored), *Item2* Random allocation, *Item3* Concealed allocation, *Item4* Baseline comparability, *Item5* Blind subjects, *Item6* Blind therapists, *Item7* Blind assessors, *Item8* Adequate follow-up, *Item9* Intention-to-treat analysis, *Item10* Between-group comparisons, *Item11* Point estimates and variability, *Y* YES, *N* NO. Note: item 1 does not contribute to total score

### Effects of interventions and the quality of the evidence

**Fig. 2:**
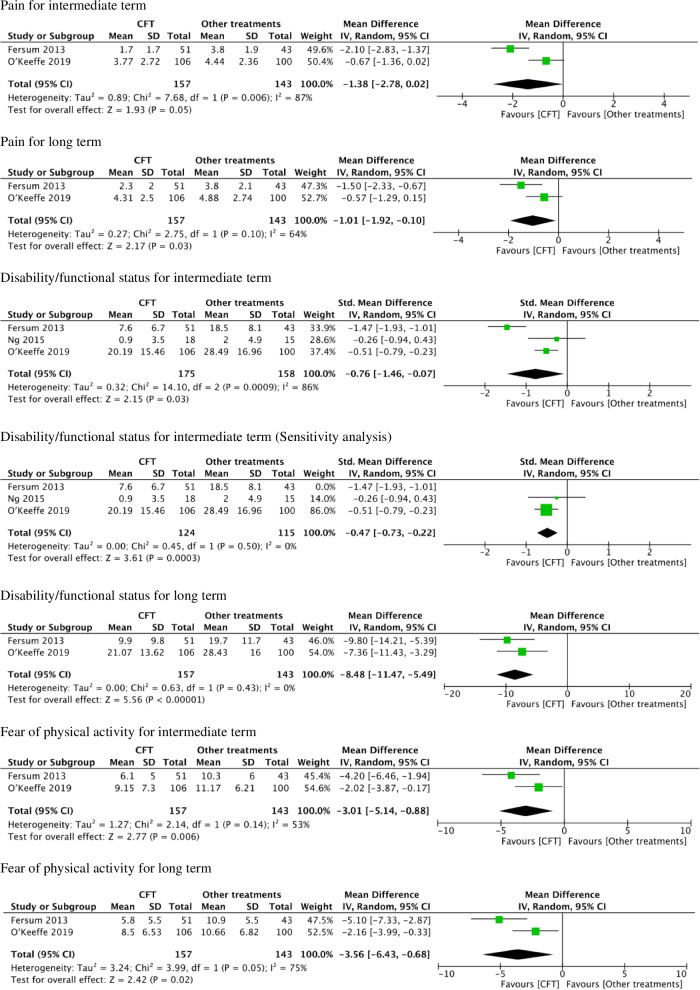
Meta-analysis

No study reported the change values from the baseline to each follow-up point. There was no response from each corresponding author, and the values at each follow-up point were used for meta-analysis (Fig. 2). Meta-analysis was performed for each outcome in the intermediate- and long-terms. In the short term, meta-analysis was not performed because only one study [21] was reported. Also, the quality of the evidence using the GRADE approach is summarized in Table 3. No disagreement was found in any rating between the two authors.

**Table 3:** A summary of the quality of the evidence using the GRADE approach

Quality assessment						Summary of findings		
No of studies	Risk of bias	Imprecision	Inconsistency	Indirectness	Publication bias	No of participants	Pooled standardised mean difference (95% confidence intervals)	Quality of evidence
Pain for intermediate term								
2	Serious risk of bias, indicating downgraded on level due to there was more than 25% of participants from studies	Very serious imprecision due to very limited sample sizes (fewer than 400 participants), and no significant difference rate down two levels Significant	Very Serious Downgraded two levels due to I^2^ > 75%, rate down two levels	Not serious Indirectness, do not downgrade	Undetected	300	-1.38 (-2.78 to 0.02)	⨁◯◯◯ VERY LOW
Pain for long term								
2	Serious risk of bias, indicating downgraded on level due to there was more than 25% of participants from studies	Very serious imprecision due to very limited sample sizes (fewer than 400 participants), and no significant difference rate down two levels Significant	Serious Downgraded one level due to I^2^ > 50%, rate down one level	Not serious Indirectness, do not downgrade	Undetected	300	-1.01 (-1.92 to -0.10)	⨁◯◯◯ VERY LOW
Disability/functional status for intermediate term								
3	Serious risk of bias, indicating downgraded on level due to there was more than 25% of participants from studies	Serious imprecision due to very limited sample sizes (fewer than 400 participants), rate down one level Significant	Very Serious Downgraded two levels due to I^2^ > 75%, rate down two levels	Serious Downgraded one level due to different sample	Undetected	333	-0.76 (-1.46 to -0.07)	⨁◯◯◯ VERY LOW
Disability/functional status for long term								
2	Serious risk of bias, indicating downgraded on level due to there was more than 25% of participants from studies	Serious imprecision due to very limited sample sizes (fewer than 400 participants), rate down one level Significant	Serious Downgraded one level due to I^2^ > 50%, rate down one level	Not serious Indirectness, do not downgrade	Undetected	300	-8.48 (-11.47 to -5.49)	⨁◯◯◯ VERY LOW
Fear of physical activity for intermediate term								
2	Serious risk of bias, indicating downgraded on level due to there was more than 25% of participants from studies	Serious imprecision due to very limited sample sizes (fewer than 400 participants), rate down one level Significant	Serious Downgraded one level due to I^2^ > 50%, rate down one level	Not serious Indirectness, do not downgrade	undetected	300	-3.01 (-5.14 to -0.88)	⨁◯◯◯ VERY LOW
Fear of physical activity for long term								
2	Serious risk of bias, indicating downgraded on level due to there was more than 25% of participants from studies	Serious imprecision due to very limited sample sizes (fewer than 400 participants), rate down one level Significant	Very Serious Downgraded two levels due to I^2^ > 75%, rate down two levels	Not serious Indirectness, do not downgrade	Undetected	300	-3.56 (-6.43 to -0.68)	⨁◯◯◯ VERY LOW

#### The CFT versus other treatments for pain intensity for the intermediate-term

Regarding pain intensity at the intermediate-term period, data from the two studies [19, 21] were synthesised. The cumulated sample size of participants in the CFT and other treatments groups was 157 and 143, respectively. A statistically significant overall effect was not detected (*P* = 0.05, MD [95% CIs] = -1.38 [-2.78 − 0.02]), indicating that the CFT is not superior to other treatments in terms of pain intensity at the intermediate-term period. The I^2^ value was 87%, indicating substantial heterogeneity. The forest plot is shown in Fig. 2. The quality of the evidence using the GRADE approach was very low (Table 3).

#### The CFT versus other treatments for pain intensity for the long-term

Regarding pain intensity at the long-term period, data from the two studies [19, 21] were synthesised. The cumulated sample size of participants in the CFT and other treatments groups was 157 and 143 participants, respectively. A statistically significant overall effect was detected (*P* = 0.03, MD [95% CIs] = -1.01 [-1.92 − -0.10]), indicating that the CFT is superior to other treatments in terms of pain intensity at the long-term period. The I^2^ value was 64%, indicating substantial heterogeneity. The forest plot is shown in Fig. 2. The quality of the evidence using the GRADE approach was very low (Table 3).

#### The CFT versus other treatments for disability/functional status for the intermediate-term

Regarding disability/functional status at the intermediate-term period, data from the three studies [19–21] were synthesised, and the forest plot is shown in Fig. 2. The cumulated sample size of participants in the CFT and other treatments groups was 175 and 158, respectively. Statistically significant overall effect was detected (*P* = 0.03, SMD [95% CIs] = -0.76 [-1.46 − -0.07]), indicating that the CFT is superior to other treatments in terms of disability/functional status at the intermediate-term period. The I^2^ value was 86%, indicating considerable heterogeneity. The quality of the evidence using the GRADE approach was very low (Table 3)**.**

Sensitivity analyses were conducted for the comparison disability/functional status at the intermediate-term period with the exception of Fersum et al.’s study (Fig. 2) [19]. A statistically significant overall effect was detected (*P* = 0.0003, SMD [95% CIs] = - 0.47 [- 0.73 − -0.22]), indicating that CFT is superior to the other treatments. No notable change was found in the statistical significance of the outcomes except the I^2^ of 0%.

#### The CFT versus other treatments for disability/functional status for the long-term

Regarding disability/functional status at the long-term period, data from the two studies [19, 21] were synthesised, and the forest plot is shown in Fig. 2. The cumulated sample size of participants in the CFT and other treatments groups was 157 and 143, respectively. A statistically significant overall effect was detected (*P* < 0.0001, MD [95% CIs] = -8.48 [-11.47 − -5.49]), indicating that the CFT is superior to other treatments in terms of disability/functional status at the long-term period. The I^2^ value was 0%, indicating insignificant heterogeneity. The quality of the evidence using the GRADE approach was very low (Table 3).

#### The CFT versus other treatments for FABQ score for the intermediate-term

Regarding the fear of physical activity at the intermediate-term period, data from the two studies [19, 21] were synthesised, and the forest plot is shown in Fig. 2. The cumulated sample size of participants in the CFT and other treatments groups was 157 and 143, respectively. Statistically significant overall effect was detected (*P* < 0.0001, MD [95% CIs] = -3.01 [-5.14 − -0.88]), indicating that the CFT is superior to other treatments in terms of fear of physical activity at the intermediate-term period. The I^2^ value was 53%, indicating moderate heterogeneity. The quality of the evidence using the GRADE approach was very low (Table 3).

#### The CFT versus other treatments for fear of physical activity for the long-term

Regarding fear of physical activity at the long-term period, data from the two studies [19, 21] were synthesised, and the forest plot is shown in Fig. 2. The cumulated sample size of participants in the CFT and other treatments groups was 157 and 143, respectively. Statistically significant overall effect was detected (*P* = 0.02, MD [95% CIs] = -3.56 [-6.43 − -0.68]), indicating that the CFT is superior to other treatments in terms of fear of physical activity at the long-term period. The I^2^ value was 75%, indicating substantial heterogeneity. The quality of the evidence using the GRADE approach was very low (Table 3).

## Discussion

To our knowledge, this is the first systematic review with the meta-analysis of RCTs examining the effect of the CFT in comparison to other treatments. A statistically significant benefit was detected in pain intensity for the long-term period, the disability/functional status and FABQ scores at the intermediate and long-term periods, but not in pain intensity for the intermediate-term period. It was also found that the quality of evidence in each meta-analysis was very low for all outcomes.

A statistically significant benefit was observed in the CFT in comparison to other treatments in the disability/functional status, but not in the pain intensity for the intermediate-term period. In addition, there was a statistically significant difference in pain intensity for the long-term period, but the effect was small. This finding may reflect the CFT characteristic, which aims to promote behavioural change and patient’s self-management through a multifaceted approach, not to improve pain intensity [7]. Further, a caution may be required when the difference in the disability/functional status is interpreted from a clinical perspective. A statistically significant difference was found, but discussions may occur considering the clinically important difference. Intra-group improvements of 12% [21] and 14% [19] on the ODI have been reported in RCTs. Although there is no universally established clinically important difference for the ODI and the RMDQ scores, Copay et al. [24] suggested the use of the minimum clinically important difference (MCID) of 14.9% for group comparison in the ODI, and 5 points were suggested by studies for the RMDQ [25–27]. The MCID validation is best used for cohort studies and may be too stringent to be applied to RCTs; however, when the threshold of 14.9% for ODI and 5 for RMDQ were used for conservative interpretations of the results, the statistically significant differences detected by the CFT compared to other treatments in disability/functional status at each follow-up point would be negligible. Therefore, it may be prudent to interpret that the effect of the CFT in pain and disability/functional status is limited for now.

A statistically significant benefit was observed in the CFT compared to other treatments in the FABQ score. Although there is no universally established MCID for the FABQ, Monticone et al. [28] reported the MCID of 4 and George et al. [29] reported 13. When these thresholds are taken into consideration, the detected statistically significant differences of the FABQ by the CFT in comparison to other treatments may not be clinically important. Further studies using a measure for fear of movement with higher responsiveness may be required in the future.

In this study, GRADE was very low in outcomes of pain intensity, as well as the other outcomes including disability/functional staus and fear of phycial activity were very low. The main reason for downgrading is the impression regarding the smaller sample size which is less than 400. Another reason was the risk of bias and inconsistency for downgrading. Furthermore, publication bias has not been assessed. The Cochrane Collaboration recommends ten or more studies to formally assess for publication bias [15]. Therefore, further RCTs are required to increase the overall evidence level of findings of the CFT effectiveness. Although the GRADE is improved with additional RCTs, there would be a barrier to strongly recommend the CFT in a clinical practice guideline, which is associated with equality for patients and feasibility in wide clinical practice. All three RCTs included in this study were undertaken by the same research group. This is because the CFT requires a certain level of knowledge and skills of the practitioner, and acquiring adequate skill to perform the CFT is not straightforward [30]. The magnitude of recommendation in a clinical practice guideline is reduced when equality for patients and feasibility are challenging [31]. Therefore, for strong recommendation of the CFT in clinical practice guidelines, a system to guarantee the skill level of the CFT must be established in the future.

Strengths of this systematic review include that this is the first summarize CFT’s effectiveness. We found that all studies were reported only by the same study group and that no RCTs existed that compared CFT with CBT. The previous study summarized the effects of Physiotherapist-delivered CBT and the results indicated that CBT group was more effective in improving pain and disability. The long-term effects were -0.21 [-0.33 to -0.09] (SMD [95% CIs]) and -0.19 [-0.32 to -0.07] (SMD [95% CIs]), respectively [5]. This was slightly smaller effects sizes than in the current study. In addition, interdisciplinary treatment based on the BPS model, provided by a multidisciplinary team, has also been shown to be effective for pain and disability. Their effects were in the long terms were 0.51 [-0.01 to 1.14] (SMD [95% CIs]) and 0.68 [0.16 to 1.19] (SMD [95% CIs]), respectively [32]. CFT had a larger effect than other BPS model-based interventions although this was not a large difference compared to the current study. It might be because the same research group that conducted the CFT intervention studied thoroughly and had sufficient pre-training when performing CFT [7]. Another reason may be that CFT is a program developed specifically for NSCLBP, while CBT and interdisciplinary treatment are broad-based for chronic pain [6, 7]. However, it is difficult to conclude which is better because CFT and CBT or interdisciplinary treatment have not been directly compared. Therefore, future studies are necessary to enhance the generalizability of the findings. For instance, one merit of CFT versus CBT is the inclusion of biomedical approach by physical therapists to satisfy comprehensibility of the BPS components. Furthermore, in this review, only the fear of physical activity was synthesized as a psychological factor. In BPS, psychosocial factors other than fear of physical activity are included, such as pain, catastrophic thinking, self-efficacy, medical costs, sick leave duration, and presenteeism. Future RCTs with a variety of psychosocial factors are required to fully understand CFT’s characteristics.

## Limitations

This study had two limitations. The first and greatest is the lack of identical treatments in the comparison group. A meta-analysis is necessary in this situation to gain specific understanding of CFT’s effectiveness with a certain intervention. Second limitation was the search strategy. Only published RCTs were included in this study. Therefore, it is possible that there are reports that were not found. However, a manual search was performed as much as possible. A cross-referencing was also done, so the conclusions are not going to change significantly. Only three RCTs were included in the meta-analysis is another limitation of this study. In the future, when more RCTs reporting the effects of CFT are available, the results of this study can be strengthened.

## Conclusion

We have very little confidence that CFT is more effective than other interventions for reducing disability in the intermediate and long-term follow-up. The true is likely to be substantially different from the estimate of effect. CFT is unique from CBT and interdisciplinary treatment in that it is led by physical therapists, who can provide physical interventions based on their expertise. However, integrating this study with previous studies, we could not conclude that CFT is superior to other BPS model-based interventions. CFT’s effectiveness must be re-evaluated in the future in larger RCTs with low risk of bias and in comparisons with identical interventions.

## Supplementary Information

**Additional file 1.** Cognitive functional therapy.

**Additional file 2.** Search strategy.

## Data Availability

Not applicable.

## Abbreviations

CFT: Cognitive functional therapy
CNSLBP: Chronic nonspecific low back pain
BPS: The Bio-Psych-Social
CBT: Cognitive − behavioral therapy
LBP: Low back pain
RCTs: Randomized controlled trials
GRADE: The Grading of Recommendations Assessment, Development and Evaluation
NRS: Numerical Rating Scale
ODI: Oswestry Disability Index
FABQ: Fear-Avoidance Beliefs Questionnaire
MDCS: Multidimensional Classification System
ROM: Range of Motion
RDQ: Roland-Morris Disability Questionnaire
HSCL-25: Hopkins Symptoms Checklist
PSQ: Patient Satisfaction Questionnaire
OMPQ: Orebro Musculoskeletal Pain Questionnaire
CNSLBP: Chronic non-specific low back pain
BBQ: Back Beliefs Questionnaire
PSEQ: Pain Self-Efficacy Questionnaire
CSQ: Coping Strategies Questionnaire
SHC: Subjective Health Complaints Inventory
DASS: Depression, Anxiety and Stress Scale
MCID: Minimum clinically important difference
MD: The mean difference
SMD: The standardised mean difference
