# Supplementary material for: The effect of cognitive functional therapy for chronic nonspecific low back pain: a systematic review and meta-analysis
Source: Biopsychosoc Med. 2022 May 21;16:12. doi: 10.1186/s13030-022-00241-6 (PMC9123771; doi:10.1186/s13030-022-00241-6)
Supplement: Supplementary file 1 — Additional file 1. Cognitive functional therapy. [file 13030_2022_241_MOESM1_ESM.docx]

Additional file 1 Cognitive Functional Therapy

Cognitive Functional Therapy (CFT) uses a multidimensional "clinical reasoning framework" to carefully listen to an individual's story based on listening and examining an individual's behavioural responses to pain, the main modifiable modifications for management Identify the target. There are mainly three components which are "Making sense of pain". "Exposure with control", and "Lifestyle change."

Making sense of Pain

- Understanding of pain based on the bio-psycho-social model
- Understanding how contextual factors, negative pain beliefs, and unhelpful emotional and behavioural responses set up a vicious cycle of pain, distress and disability.
- It was discussed collaboratively and reflectively.
- clear and realistic self-motivated strategies for behavioural change directed to their personally relevant goals are identified
- Online resources and patient stories are also provided to facilitate this learning process.

Exposure with control

- A process of behavioural change through experiential learning
- It consists of "functional movement training" and "functional integration".
- It is designed to challenge expectations of pain and damage outcomes through guided behavioural experiments.
- It involves sympathetic nervous system responses that manifest during exposure to painful, feared, or avoided functional tasks (rapid supratentorial breathing and body tension) and safety-seeking behaviours (protective muscle guards, breath-holds, movement avoidance and hand props) that are explicitly targeted and controlled.
- Targeted functional conditioning is used when there is a lack of strength or endurance, which is an obstacle to achieving personal goals.
- Emphasis on hands-on feedback should be a minimal part of the session.
- The new strategy is immediately integrated into goal-oriented daily activities to build self-efficacy and body conditioning.

Lifestyle change

- This involves behavioural modification addressing unhelpful lifestyle factors aimed at increasing physical activity levels.
- For example, sleep disorders and disorders can be addressed in a variety of ways, based on how they affect the pain experience of the people identified during the interview or examination.
- Sleep disturbances due to pain, worry, or stress can be addressed by exploring the effects of body relaxation, breathing regulation, guided meditation techniques, and engaging in physical activity.
