# Supplementary material for: The effect of cognitive functional therapy for chronic nonspecific low back pain: a systematic review and meta-analysis
Source: Biopsychosoc Med. 2022 May 21;16:12. doi: 10.1186/s13030-022-00241-6 (PMC9123771; doi:10.1186/s13030-022-00241-6)
Supplement: Supplementary file 2 — Additional file 2. Search strategy. [file 13030_2022_241_MOESM2_ESM.docx]

Additional file 2 Search Strategy

Database: Ovid MEDLINE(R) and Epub Ahead of Print, In-Process & Other Non-Indexed Citations and Daily <1946 to Dec 30, 2020>

Search Strategy:

--------------------------------------------------------------------------------

1 Chronic Pain/ (13616)

2 Musculoskeletal Pain/ (3069)

3 Back Pain/ (17405)

4 (musculoskeletal pain or lumber vertebrae or back pain or lumbago or backache or back ache).tw. (53594)

5 (back adj3 disorder).tw. (244)

6 (low$ adj3 back adj3 pain).tw. (29606)

7 Cognitive Behavioral Therapy/ (24657)

8 cognitive modality therapy.tw. (0)

9 cognitive functional therapy.tw. (26)

10 (cogni$ adj therap$).tw. (3023)

11 (biopsychosocial or multidisciplinary or multiprofession$ or interdisciplina$ or multimodal).tw. (149656)

12 randomized controlled trial.mp. (527576)

13 controlled clinical trial.mp. (107189)

14 randomized.mp. [mp=title, abstract, original title, name of substance word, subject heading word, floating sub-heading word, keyword heading word, organism supplementary concept word, protocol supplementary concept word, rare disease supplementary concept word, unique identifier, synonyms] (826029)

15 placebo.mp. [mp=title, abstract, original title, name of substance word, subject heading word, floating sub-heading word, keyword heading word, organism supplementary concept word, protocol supplementary concept word, rare disease supplementary concept word, unique identifier, synonyms] (211835)

16 drug therapy.mp. [mp=title, abstract, original title, name of substance word, subject heading word, floating sub-heading word, keyword heading word, organism supplementary concept word, protocol supplementary concept word, rare disease supplementary concept word, unique identifier, synonyms] (2261621)

17 randomly.mp. [mp=title, abstract, original title, name of substance word, subject heading word, floating sub-heading word, keyword heading word, organism supplementary concept word, protocol supplementary concept word, rare disease supplementary concept word, unique identifier, synonyms] (328311)

18 trial.mp. [mp=title, abstract, original title, name of substance word, subject heading word, floating sub-heading word, keyword heading word, organism supplementary concept word, protocol supplementary concept word, rare disease supplementary concept word, unique identifier, synonyms] (1168808)

19 groups.mp. [mp=title, abstract, original title, name of substance word, subject heading word, floating sub-heading word, keyword heading word, organism supplementary concept word, protocol supplementary concept word, rare disease supplementary concept word, unique identifier, synonyms] (2145560)

20 humans.mp. [mp=title, abstract, original title, name of substance word, subject heading word, floating sub-heading word, keyword heading word, organism supplementary concept word, protocol supplementary concept word, rare disease supplementary concept word, unique identifier, synonyms] (18435463)

21 1 or 2 or 3 or 4 or 5 or 6 (75018)

22 7 or 8 or 9 or 10 or 11 (174657)

23 12 or 13 or 14 or 15 or 16 or 17 or 18 or 19 (5015190)

24 20 and 23 (3842969)

25 21 and 22 and 24 (1361)

***************************

Database: Embase Classic+Embase <1947 to 2020 Dec 30>

Search Strategy:

--------------------------------------------------------------------------------

1 exp chronic pain/ (59731)

2 exp musculoskeletal pain/ (149389)

3 exp backache/ (110336)

4 (musculoskeletal pain or lumber vertebra or back pain or lubago or backache or back ache).tw. (76258)

5 (back adj3 disorder).tw. (317)

6 (low$ adj3 back adj3 pain$).tw. (41284)

7 cognitive behavioral therapy/ (10918)

8 cognitive modality therapy.tw. (0)

9 cognitive functinal therapy.tw. (0)

10 (cogni$ adj therap$).tw. (4869)

11 (biopsychosocial or multidisciplinary or multiprofession$ or interdisciplina$ or multimodal).tw. (229881)

12 randomized controlled trial.mp. [mp=title, abstract, heading word, drug trade name, original title, device manufacturer, drug manufacturer, device trade name, keyword, floating subheading word, candidate term word] (782074)

13 controlled clinical trial.mp. [mp=title, abstract, heading word, drug trade name, original title, device manufacturer, drug manufacturer, device trade name, keyword, floating subheading word, candidate term word] (485199)

14 randomized.mp. [mp=title, abstract, heading word, drug trade name, original title, device manufacturer, drug manufacturer, device trade name, keyword, floating subheading word, candidate term word] (1089933)

15 placebo.mp. [mp=title, abstract, heading word, drug trade name, original title, device manufacturer, drug manufacturer, device trade name, keyword, floating subheading word, candidate term word] (458488)

16 drug therapy.mp. [mp=title, abstract, heading word, drug trade name, original title, device manufacturer, drug manufacturer, device trade name, keyword, floating subheading word, candidate term word] (4555100)

17 randomly.mp. [mp=title, abstract, heading word, drug trade name, original title, device manufacturer, drug manufacturer, device trade name, keyword, floating subheading word, candidate term word] (437853)

18 trial.mp. [mp=title, abstract, heading word, drug trade name, original title, device manufacturer, drug manufacturer, device trade name, keyword, floating subheading word, candidate term word] (2125295)

19 groups.mp. [mp=title, abstract, heading word, drug trade name, original title, device manufacturer, drug manufacturer, device trade name, keyword, floating subheading word, candidate term word] (2986340)

20 humans.mp. [mp=title, abstract, heading word, drug trade name, original title, device manufacturer, drug manufacturer, device trade name, keyword, floating subheading word, candidate term word] (481838)

21 1 or 2 or 3 or 4 or 5 or 6 (211872)

22 7 or 8 or 9 or 10 or 11 (244657)

23 21 and 22 (8176)

24 12 or 13 or 14 or 15 or 16 or 17 or 18 or 19 (8225510)

25 20 and 24 (117461)

26 23 and 25 (25)

***************************

Database: Emcare　<1806 to Dec Week 4 2020>

Search Strategy:

--------------------------------------------------------------------------------

1 exp chronic pain/ (59731)

2 exp musculoskeletal pain/ (149389)

3 exp backache/ (110336)

4 (musculoskeletal pain or lumber vertebra or back pain or lubago or backache or back ache).tw. (76258)

5 (back adj3 disorder).tw. (317)

6 (low$ adj3 back adj3 pain$).tw. (41284)

7 cognitive behavioral therapy/ (10918)

8 cognitive modality therapy.tw. (0)

9 cognitive functinal therapy.tw. (0)

10 (cogni$ adj therap$).tw. (4869)

11 (biopsychosocial or multidisciplinary or multiprofession$ or interdisciplina$ or multimodal).tw. (229881)

12 randomized controlled trial.mp. [mp=title, abstract, heading word, drug trade name, original title, device manufacturer, drug manufacturer, device trade name, keyword, floating subheading word, candidate term word] (782074)

13 controlled clinical trial.mp. [mp=title, abstract, heading word, drug trade name, original title, device manufacturer, drug manufacturer, device trade name, keyword, floating subheading word, candidate term word] (485199)

14 randomized.mp. [mp=title, abstract, heading word, drug trade name, original title, device manufacturer, drug manufacturer, device trade name, keyword, floating subheading word, candidate term word] (1089933)

15 placebo.mp. [mp=title, abstract, heading word, drug trade name, original title, device manufacturer, drug manufacturer, device trade name, keyword, floating subheading word, candidate term word] (458488)

16 drug therapy.mp. [mp=title, abstract, heading word, drug trade name, original title, device manufacturer, drug manufacturer, device trade name, keyword, floating subheading word, candidate term word] (4555100)

17 randomly.mp. [mp=title, abstract, heading word, drug trade name, original title, device manufacturer, drug manufacturer, device trade name, keyword, floating subheading word, candidate term word] (437853)

18 trial.mp. [mp=title, abstract, heading word, drug trade name, original title, device manufacturer, drug manufacturer, device trade name, keyword, floating subheading word, candidate term word] (2125295)

19 groups.mp. [mp=title, abstract, heading word, drug trade name, original title, device manufacturer, drug manufacturer, device trade name, keyword, floating subheading word, candidate term word] (2986340)

20 humans.mp. [mp=title, abstract, heading word, drug trade name, original title, device manufacturer, drug manufacturer, device trade name, keyword, floating subheading word, candidate term word] (481838)

21 1 or 2 or 3 or 4 or 5 or 6 (211872)

22 7 or 8 or 9 or 10 or 11 (244657)

23 21 and 22 (8176)

24 12 or 13 or 14 or 15 or 16 or 17 or 18 or 19 (8225510)

25 20 and 24 (117461)

26 23 and 25 (25)

***************************

Database: AMED (Allied and Complementary Medicine) <1985 to Dec 2020>

Search Strategy:

--------------------------------------------------------------------------------

1 Pain/ (12491)

2 Musculoskeletal Pain/ (226)

3 Back Pain/ or low back pain/ or backache.mp. [mp=abstract, heading words, title] (6618)

4 (musculoskeletal pain or lumber vertebrae or back pain or lumbago or backache or back ache).tw. (8368)

5 (back adj3 disorder).tw. (51)

6 (low$ adj3 back adj3 pain).tw. (6039)

7 Cognitive Therapy/ (1339)

8 cognitive modality therapy.tw. (0)

9 cognitive functional therapy.tw. (7)

10 (cogni$ adj therap$).tw. (1401)

11 (biopsychosocial or multidisciplinary or multiprofession$ or interdisciplina$ or multimodal).tw. (4104)

12 randomized controlled trial.mp. (3264)

13 controlled clinical trial.mp. (540)

14 randomized.mp. [mp=abstract, heading words, title] (12064)

15 placebo.mp. [mp=abstract, heading words, title] (3295)

16 drug therapy.mp. [mp=abstract, heading words, title] (27831)

17 randomly.mp. [mp=abstract, heading words, title] (6354)

18 trial.mp. [mp=abstract, heading words, title] (11654)

19 groups.mp. [mp=abstract, heading words, title] (26547)

20 humans.mp. [mp=abstract, heading words, title] (140082)

21 1 or 2 or 3 or 4 or 5 or 6 (20188)

22 7 or 8 or 9 or 10 or 11 (5453)

23 12 or 13 or 14 or 15 or 16 or 17 or 18 or 19 (64841)

24 20 and 23 (31233)

25 21 and 22 and 24 (132)

Database: PsycINFO <1806 to Dec Week 4 2020>

Search Strategy:

--------------------------------------------------------------------------------

1 exp Chronic Pain/ (13123)

2 exp Musculoskeletal Disorders/ (17967)

3 exp Back Pain/ (3811)

4 (musculoskeletal pain or lumber verebrase or back pain or lumbago or backache or back ache).tw. (7009)

5 (back adj3 disorder).tw. (69)

6 (low$ adj3 back adj3 pain$).tw. (3851)

7 exp Cognitive Behavior Therapy/ (20838)

8 cognitive modality therapy.tw. (0)

9 cognitive functinal therapy.tw. (0)

10 (cogni$ adj therap$).tw. (7614)

11 (biopsychosocial or multidisciplinary or multiprofession$ or interdisciplina$ or multimodal).tw. (59311)

12 randomized controlled trial.mp. [mp=title, abstract, heading word, table of contents, key concepts, original title, tests & measures, mesh] (17661)

13 controlled clinical trial.mp. [mp=title, abstract, heading word, table of contents, key concepts, original title, tests & measures, mesh] (1506)

14 randomized.mp. [mp=title, abstract, heading word, table of contents, key concepts, original title, tests & measures, mesh] (72828)

15 placebo.mp. [mp=title, abstract, heading word, table of contents, key concepts, original title, tests & measures, mesh] (39859)

16 drug therapy.mp. [mp=title, abstract, heading word, table of contents, key concepts, original title, tests & measures, mesh] (137183)

17 randomly.mp. [mp=title, abstract, heading word, table of contents, key concepts, original title, tests & measures, mesh] (71480)

18 trial.mp. [mp=title, abstract, heading word, table of contents, key concepts, original title, tests & measures, mesh] (106164)

19 groups.mp. [mp=title, abstract, heading word, table of contents, key concepts, original title, tests & measures, mesh] (532218)

20 humans.mp. [mp=title, abstract, heading word, table of contents, key concepts, original title, tests & measures, mesh] (1237906)

21 1 or 2 or 3 or 4 or 5 or 6 (34175)

22 7 or 8 or 9 or 10 or 11 (85833)

23 12 or 13 or 14 or 15 or 16 or 17 or 18 or 19 (794269)

24 20 and 23 (296450)

25 21 and 22 and 24 (418)

Web of science

TS= (((Chronic OR musculoskeletal OR low*) NEAR/3 (back or lumbago) NEAR/3 (pain* OR disorder OR ache)) AND ((cogni* NEAR therap*) OR (biopsychosocial OR multidisciplinary OR multiprofession* OR interdisciplina* OR multimodal)))

Search Name: CENTRAL

Last Saved: 30/12/2020 06:39:11

Comment:

ID Search

#1 MeSH descriptor: [Chronic Pain] explode all trees

#2 MeSH descriptor: [Musculoskeletal Pain] explode all trees

#3 MeSH descriptor: [Back Pain] explode all trees

#4 (musculoskeletal pain OR lumber vertebrae OR back pain OR lumbago OR backache OR back ache):ti,ab,kw

#5 {OR #1-#4}

#6 MeSH descriptor: [Cognitive Behavioral Therapy] explode all trees

#7 (cognitive modality therapy):ti,ab,kw

#8 (cognitive functional therapy):ti,ab,kw

#9 (cogni* NEAR therap*):ti,ab,kw

#10 (biopsychosocial OR multidisciplinary OR multiprofession* OR interdisciplina* OR multimodal):ti,ab,kw

#11 {OR #6-#10}

#12 #5 and #11

#13 (Randomized controlled trial):ti,ab,kw

#14 (controlled clinical trial):ti,ab,kw

#15 {OR #13-#14}

#16 (humans):ti,ab,kw

#17 #15 and #16

#18 #12 and #17

Database: CINAHL plus ( EBSCOhost interface )

Search Strategy:

--------------------------------------------------------------------------------

S1 (MH "Chronic Pain")

S2 "Musculoskeletal Pain"

S3 (MH "Back Pain+")

S4 (musculoskeletal pain or lumber vertebrae or back pain or lumbago or backache or back ache)

S5 (back adj3 disorder)

S6 (low$ adj3 back adj3 pain)

S7 "Cognitive Behavioral Therapy"

S8 cognitive modality therapy

S9 cognitive functional therapy

S10 (biopsychosocial or multidisciplinary or multiprofession or interdisciplina or multimodal)

S11 randomized controlled trial

S12 controlled clinical trial

S13 randomized

S14 placebo

S15 drug therapy

S16 randomly

S17 trial

S18 groups

S19 humans

S20 S1 OR S2 OR S3 OR S4 OR S5 OR S6

S21 S7 OR S8 OR S9 OR S10

S22 S11 OR S12 OR S13 OR S14 OR S15 OR S16 OR S17 OR S18

S23 S19 AND S22

S24 S20 AND S21 AND S23

***************************

Physiotherapy Evidence Database (PEDro) (available at pedro.org.au)

Abstract & Title: cogni*

Body Part: lumber spine, sacro-iliac joint or pelvis

Method: clinical trial
